# A Feature Integration Network for Multi-Channel Speech Enhancement

**DOI:** 10.3390/s24227344

**Published:** 2024-11-18

**Authors:** Xiao Zeng, Xue Zhang, Mingjiang Wang

**Affiliations:** Key Laboratory for Key Technologies of IoT Terminals, Harbin Institute of Technology, Shenzhen 518055, China; zengxiao0106@163.com (X.Z.); 13115501669@163.com (X.Z.)

**Keywords:** multi-channel speech enhancement, LSTM, deep learning, self-attention

## Abstract

Multi-channel speech enhancement has become an active area of research, demonstrating excellent performance in recovering desired speech signals from noisy environments. Recent approaches have increasingly focused on leveraging spectral information from multi-channel inputs, yielding promising results. In this study, we propose a novel feature integration network that not only captures spectral information but also refines it through shifted-window-based self-attention, enhancing the quality and precision of the feature extraction. Our network consists of blocks containing a full- and sub-band LSTM module for capturing spectral information, and a global–local attention fusion module for refining this information. The full- and sub-band LSTM module integrates both full-band and sub-band information through two LSTM layers, while the global–local attention fusion module learns global and local attention in a dual-branch architecture. To further enhance the feature integration, we fuse the outputs of these branches using a spatial attention module. The model is trained to predict the complex ratio mask (CRM), thereby improving the quality of the enhanced signal. We conducted an ablation study to assess the contribution of each module, with each showing a significant impact on performance. Additionally, our model was trained on the SPA-DNS dataset using a circular microphone array and the Libri-wham dataset with a linear microphone array, achieving competitive results compared to state-of-the-art models.

## 1. Introduction

Multi-channel speech enhancement, which aims to recover desired speech signals in noisy environments, plays an essential role in the development of devices with multiple microphones, such as hearing aids, mobile phones, and cameras. In recent years, many researchers have focused on studying multi-channel speech enhancement. Traditional speech enhancement methods, e.g., minimum variance distortionless response beamformers (MVDR) [1], mainly leverage spatial information to suppress noise and undesired speakers. However, traditional speech enhancement methods are typically grounded in idealized assumptions, achieving strong performance with stationary noise but often struggling to effectively handle non-stationary noise.

Over recent decades, deep learning has demonstrated substantial efficacy in the field of speech enhancement [2]. This technology has the capability to autonomously extract meaningful features from spatial and spectral information, facilitating the restoration of clear target signals from noisy environments. Early studies involved the use of deep neural networks (DNNs) for time–frequency (T-F) masking, an approach inspired by the auditory masking phenomenon. This phenomenon occurs when a quieter sound becomes imperceptible in the presence of a louder sound within a critical frequency band [3]. Early research trained long short-term memory (LSTM) neural networks to generate ideal ratio masks (IRMs) for estimating the spatial covariance of noise, which were subsequently used to derive minimum variance distortionless response (MVDR) filters [4,5,6]. An IRM enhances the magnitude response of noisy speech but directly utilizes the noisy phase for reconstruction, without considering phase refinement. To address this limitation, recent studies have advanced speech enhancement through the use of complex ratio masking [7,8,9] or neural beamforming techniques. These methods have demonstrated outstanding performance and are increasingly becoming the dominant approaches in multi-channel speech enhancement.

To date, many studies have adopted encoder–decoder architectures to effectively learn hierarchical and spatial features [10,11,12]. Although stacked convolutional layers tend to incur a considerable parameter overhead, thereby increasing memory demands, LSTMs are advantageous for handling long sequences with lower memory requirements, enhancing their effectiveness for temporal context learning. RNN-based approaches employ a series of RNN blocks to model sequences across both frequency and time dimensions [7,8,13,14]. Li’s team proposed a full-band and sub-band fusion model to learn global and local spectral patterns for single-channel speech enhancement [15]. Subsequently, they further leveraged spatial information for multi-channel speech enhancement by designing a fused multiple-cue network, capable of fully exploiting both spectral and spatial information [8]. Meanwhile, Kristina et al. proposed the FT-JNF network [7], which allows control over the availability of spatial, spectral, and temporal information, allowing for the analysis of the interdependencies within temporal and spectral domains. The FT-JNF network comprises two stacked LSTM layers that separately process full-band and sub-band data. Furthermore, Wang et al. integrated full- and sub-band modeling with LSTM-based approaches to reduce algorithmic complexity and minimize latency [13,14]. These methods demonstrated the effectiveness of full-band and sub-band fusion models in speech enhancement tasks. Accordingly, the model proposed herein also incorporates full- and sub-band modules to capture both spatial and spectral information.

Transformers [16] and their variants have been extensively utilized in speech enhancement [9,17,18], due to their ability to model global information. On the other hand, models based on convolutional neural networks (CNNs) excel at capturing detailed local contextual information [19]. Various strategies exist for integrating CNNs and transformers to effectively exploit both global and local features, thereby leveraging the strengths of each architecture. For instance, Xiang et al. [20] introduced a multiscale aggregation block that integrates local and global speech features synergistically. However, the standard self-attention architecture requires significant computational time and memory resources to effectively capture the global context.

The aforementioned methods have demonstrated that employing stacked LSTMs as the DNN backbone to integrate full- and sub-band information yields impressive performance. Given that LSTMs are advantageous for handling long sequences with reduced memory requirements, we employ stacked LSTMs as the DNN backbone to effectively process these sequences, while minimizing memory overhead. We propose a novel feature integration network that not only captures spectral information but also refines it through shifted-window-based self-attention, thereby enhancing the quality and precision of feature extraction. Our network consists of blocks that contain a full- and sub-band LSTM module for capturing spectral information, as well as a global–local attention fusion module for refining this information. The full- and sub-band LSTM module integrates both full-band and sub-band information through two LSTM layers. The GLAF module’s dual-branch architecture is designed to simultaneously learn global and local attention information, with a spatial attention (SA) module further refining this integration. Unlike traditional methods, the global branch of our model incorporates an efficient global–local attention mechanism [21] to effectively capture global information, while the local branch leverages convolutional layers for precise local feature extraction. Additionally, inspired by [22], our SA module facilitates advanced feature integration, dynamically blending global and local attention insights for optimal enhancement. Our model’s output is trained to predict the complex ratio mask, a technique that ensures more accurate signal reconstruction. To thoroughly evaluate the model, we conducted an ablation study on the SPA-DNS dataset, which uses a circular microphone array, to assess the individual contributions of each module. Furthermore, we developed the Libri-wham dataset with a linear microphone array. Our approach demonstrated competitive performance on both the SPA-DNS and Libri-wham datasets, performing favorably compared to leading models in the field.

The remainder of this paper is organized as follows: Section 2 introduces the signal model for multi-channel signal processing. Section 3 describes the proposed algorithms. Section 4 outlines the datasets and experimental setup used in our study. Section 5 presents the performance evaluation of the proposed system. Finally, Section 6 concludes the paper.

## 2. Signal Model

Considering a mixture recorded under anechoic conditions using M microphones, a physical model in the short-time Fourier transform (STFT) domain can be formulated as:

(1) $$\begin{array}{cc} y_{m}\left(f,t\right) & =s_{m}\left(f,t\right)+z_{m}\left(f,t\right)\\ & =s_{m}^{d}\left(f,t\right)+s_{m}^{r}\left(f,t\right)+z_{m}\left(f,t\right)\\ & =s_{m}^{d}\left(f,t\right)+n_{m}\left(f,t\right), \end{array}$$

where $s_{m}\left(f,t\right)$ represents the speech received at microphone *m*, which includes direct-path speech $s_{m}^{d}\left(f,t\right)$ and reverberated speech $s_{m}^{r}\left(f,t\right)$. Additionally, $z_{m}\left(f,t\right)$ denotes the noise signal at the *m*-th microphone, while $n_{m}\left(f,t\right)$ represents the overall interference, including both noise and reverberation. For brevity, the frequency and time frame indices, *f* and *t*, will be omitted in the following text. In this work, our goal is to estimate the direct-path clean speech from the reference microphone using the mixture signals captured by the microphone array. We designate the first microphone as the reference microphone.

## 3. Proposed Algorithms

**Figure 1:**
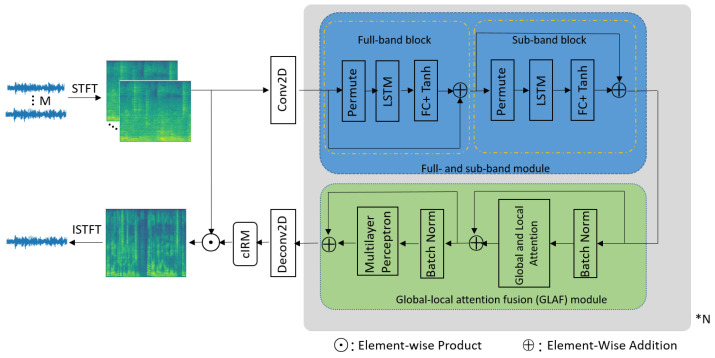
This diagram illustrates our proposed feature integration network. This architecture comprises multiple feature integration blocks, each containing a full- and sub-band module (the blue box) coupled with a global–local attention fusion module (the green box). * N means repeat the integration block (the gray box) N times.

The architecture of our system is illustrated in Figure 1. The multi-channel input signals, denoted as Noisy, are transformed into complex features via the STFT, resulting in a three-dimensional tensor $Y=\left[y_{1},\ldots ,y_{M}\right]\in C^{2M\times F\times T}$. This process involves stacking the real (R) and imaginary (I) components of the STFT in the channel dimension to form a complete representation. Here, F denotes the number of frequency bins and T denotes the number of frames. The stacked tensor is then fed into a two-dimensional (2D) convolutional (Conv2D) layer to generate an embedding. This embedding is gradually refined through *N* feature integration blocks, each consisting of a full- and sub-band LSTM (FaS) module coupled with a GLAF module. Each module incorporates a residual connection. Following this refinement, a 2D deconvolution (Deconv2D) layer is employed to predict the real and imaginary components of the complex ratio mask. The output spectrum is derived by multiplying the noisy spectrum from the reference channel with the complex ratio mask. Finally, the spectrum is transformed back into the time domain using an inverse short-time Fourier transform (ISTFT) to produce the enhanced signal. The above process can be expressed as follows:

(2) $$\begin{array}{cc} Y & =STFT(Noisy), \end{array}$$

(3) $$\begin{array}{cc} Embedding & =Conv2D(Y), \end{array}$$

(4) $$\begin{array}{cc} Refined\_feature & =GLAF(FaS(Embedding)), \end{array}$$

(5) $$\begin{array}{cc} CRM & =Deconv2D(refined\_feature), \end{array}$$

(6) $$\begin{array}{cc} Enhanced\_speech & =ISTFT(CRM*Y). \end{array}$$

The FaS module captures fine-grained spectral information, thereby enhancing the speech clarity by emphasizing key spectral features. Additionally, the GLAF module captures both global and local attention simultaneously, facilitating more effective feature learning and bolstering the model’s robustness to diverse noise conditions. A spatial attention mechanism is incorporated within the GLAF module to further refine performance, avoiding the limitations of simple concatenation or summation of global and local attention, which can impair the discriminative capacity of deep models. The integration of these modules leads to improved performance by ensuring that the model not only captures detailed spectral information (via FaS) but also learns how to prioritize and refine features based on both local and global contexts (via GLAF and spatial attention). The contributions of these components are analyzed in detail in the Experimental Setup section.

### 3.1. Full- and Sub-Band LSTM Module

A full-band and sub-band fusion model was initially proposed by [15] for single-channel speech enhancement, inspiring subsequent extensions to multi-channel speech enhancement, as seen in [7,8,13]. The blue box in Figure 1 illustrates the full- and sub-band LSTM module developed in our work. The *D*-dimensional embeddings $\tilde{D}$ obtained from the Conv2D layer are first permuted to the order (F,B,T,D), where *B* is the batch size, and then reshaped to (F,B∗T,D). These data are fed into the first LSTM layer, followed by a feedforward layer and a tanh activation function. The LSTM with $H_{1}$ hidden units models the *D*-dimensional frame embeddings, producing a tensor with shape ($F,B*T,H_{1}$). A linear layer then maps the $H_{1}$-dimensional embeddings back to *D*-dimensions, followed by a tanh activation function. This series of operations strategically focuses on full-band spatial information, enabling the network to effectively enhance and refine spatial details across the frequency spectrum. Next, the output of the full-band block is reshaped to (F,B,T,D) and added to the *D*-dimensional embeddings via a residual connection. The enhanced tensor is permuted to the order (T,B,F,D) and reshaped to (T,B∗F,D), which is then fed into the second LSTM layer. This is followed by another feedforward layer and a tanh activation function, concentrating on sub-band spatial information. The LSTM with $H_{2}$ hidden units models the *D*-dimensional frame embeddings, resulting in a tensor with shape ($F,B*T,H_{2}$). A linear layer then maps the $H_{2}$-dimensional embeddings back to *D*-dimensions, followed by a tanh activation function. Finally, the output of the sub-band block is reshaped to (F,B,T,D) and reintegrated with the input tensor of the sub-band block via a residual connection, preserving and enhancing the continuity of both spatial and spectral information. The above process can be expressed as

(7) $$\begin{array}{cc} {\tilde{B}}_{full} & =tanh\left(FC\left(LSTM_{1}\left(\tilde{D}\right)\right)\right)+\tilde{D}, \end{array}$$

(8) $$\begin{array}{cc} {\tilde{B}}_{sub} & =tanh(FC(LSTM_{2}\left({\tilde{B}}_{full}))\right)+{\tilde{B}}_{full}. \end{array}$$

### 3.2. Global–Local Attention Fusion Module

**Figure 2:**
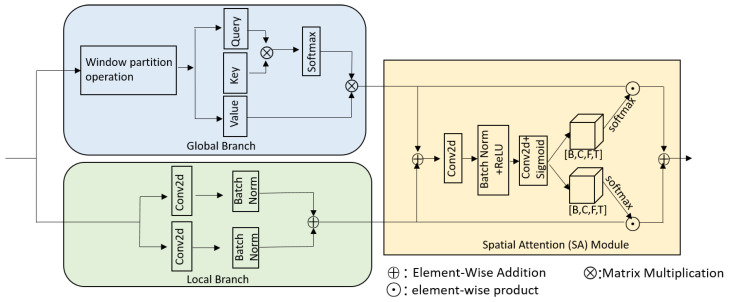
Diagram of the global and local attention fusion layer. It comprises two branches, a global branch and a local branch, along with a spatial attention (SA) module.

The green box in Figure 1 illustrates the GLAF module developed in our work. It consists of a batch normalization layer, a global and local attention fusion layer, another batch normalization layer, and a multilayer perceptron. The global and local attention fusion layer, illustrated in Figure 2, consists of two branches, a global branch and a local branch, along with a spatial attention (SA) module. The global branch captures global information, while the local branch captures local information. The local branch employs two parallel Conv2D layers, each followed by a batch normalization layer. These two Conv2D layers utilize different kernel sizes to extract local information:

(9) $$\begin{array}{c} {Output}_{local}=BN\left({Conv2D}_{1}\left({\tilde{B}}_{sub}\right)\right)+BN\left({Conv2D}_{2}\left({\tilde{B}}_{sub}\right)\right). \end{array}$$

**Figure 3:**
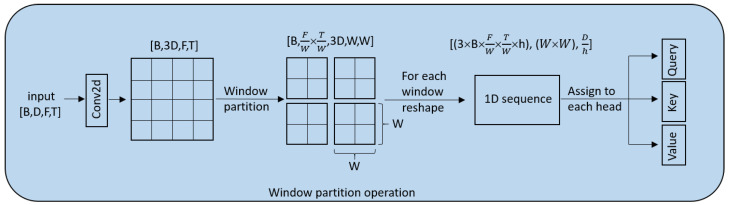
The window partition operation.

The global branch utilizes window-based multi-head self-attention [21], known for its efficiency in capturing global information. The input tensor first undergoes a window partition operation, as detailed in Figure 3. Initially, a Conv2D layer expands the channel dimension threefold. Subsequently, the feature map is divided into smaller windows. Each window is reshaped from $\left[B,\frac{F}{W},\frac{T}{W},3D,W,W\right]$ to $\left[3*B*\frac{F}{W}*\frac{T}{W}*h,W*W,\frac{D}{h}\right]$, and then split into value, key, and query vectors, which are critical for the attention mechanism. Here, *W* represents the window size and *h* represents the number of heads. The attention weights are calculated by first taking the matrix product of the key and query vectors. This is then scaled by $\sqrt{d}$, where d=D/h, to avoid large values in the matrix product which could slow down the gradient convergence. The scaled result is passed through a softmax function to obtain the normalized attention weights. These weights represent the importance of each element in the sequence relative to others within the window. Finally, the attention weights are used to compute a matrix product with the value vectors, followed by a transformation to restore the original shape (B,D,F,T). The global branch’s output is expressed mathematically as

(10) $$\begin{array}{c} {Output}_{global}=Attention\left(Q,K,V\right)=softmax\left(\frac{{\mathrm{QK}}^{T}}{\sqrt{d}}\right)V, \end{array}$$

where *T* denotes the transpose operation, and **Q, K, V** denotes the value, key, and query vectors. More details on the window-based multi-head self-attention can be found in [21].

### 3.3. Spatial Attention Module

In [23], the authors argued that simply concatenating or summing multiscale features can impair the discriminative ability of deep models. Here, we integrate ${Output}_{local}$ and ${Output}_{global}$ to enhance the performance using the SA module, rather than simply summing them, as illustrated in the yellow box in Figure 2. The spatial attention mechanism enables neurons to gather multi-scale spatial information [22]. We refine the sum of ${Output}_{local}$ and ${Output}_{global}$ using a Conv2D layer, a BatchNormalization layer, and a ReLU layer, yielding the intermediate result *p*. We then employ another Conv2D layer, followed by a sigmoid operation, to generate the weighted feature map, which is then split into $s_{1}$ and $s_{2}$. Each of these is multiplied by its respective branch output, either Outputlocal or Outputglobal. Finally, these two outputs are added to fuse the channel and spatial features, resulting in the output of the SA module, denoted as *U*. The process is outlined below:

(11) $$\begin{array}{cc} \; & p=ReLU(BN\left(Conv2D\left({Output}_{local}+{Output}_{global}\right)\right)) \end{array}$$

(12) $$\begin{array}{cc} \; & s_{1},s_{2}=Split\left(Sigmoid\left(Conv2D\left(p\right)\right)\right) \end{array}$$

(13) $$\begin{array}{cc} \; & U=s_{1}*{Output}_{local}+s_{2}*{Output}_{global} \end{array}$$

## 4. Experimental Setup

### 4.1. Dataset

In this paper, we used the Python toolbox Pyroomacoustics [24] to simulate real spatial scenarios. Considering the variety of microphone arrays in real-world applications, we created two spatial datasets with different microphone arrays to evaluate the proposed model: the SPA-DNS dataset with a circular microphone array and the Libri-wham dataset with a linear microphone array. Both the SPA-DNS and Libri-wham datasets encompass a wide range of acoustic scenarios, covering the two main types of microphone array configurations commonly used in real-world applications. We employed a method similar to that used in [25] to generate multi-channel datasets. Descriptions of these two datasets are provided below.

- The SPA-DNS dataset: This dataset was created with simulated room sizes ranging from $5\times 5\times 3\;m^{3}$ to $10\times 10\times 4\;m^{3}$, covering common indoor dimensions. Reverberation times (RT60) were varied between 0.2 and 1.2 s, simulating a range from low to moderate reverberant conditions typical in indoor environments. A circular microphone array of four microphones, with a radius of 10 cm, was randomly placed in each room. Both the array and the two sources—clean speech and noise—were positioned at random locations at least 0.5 m from the walls, with a source–source distance of 0.75 to 2 m. Clean speech and noise samples were sourced from the DNS Challenge 2020 corpus [26]. Noise clips were selected from Audioset and Freesound, encompassing a wide range of typical real-world noise types commonly encountered in daily life. In total, we generate 85,000 training utterances (3–6 s), 4400 validation utterances (3–10 s), and 2700 test utterances (3–10 s) with signal-to-noise ratios (SNRs) between −5 dB and 10 dB, mimicking real-world noisy conditions. The ratios of the training, validation, and test sets were 92%, 5%, and 3%, respectively.
- The Libri-wham dataset: The rooms were created with sizes ranging from $3\times 3\times 2.5\;m^{3}$ to $8\times 8\times 3\;m^{3}$, covering common indoor dimensions. The RT60 values ranged from 0.3 to 0.6 s, simulating a range from low to moderate reverberant conditions typical in indoor environments. A linear array of four microphones was randomly positioned within each room, with a spacing of 0.5 cm between each microphone. The two sources, consisting of speech and noise, were randomly positioned within the room at heights between 1.2 and 1.6 m, to simulate typical speaking heights and realistic noise scenarios in daily life. Clean speech files were obtained from Librispeech (train-clean-100) [27], while noise files were selected from the MUSAN dataset [28], which contains 929 diverse noise samples representing various real-world noise types encountered in everyday environments. We generated 16,724 training utterances of 6 s each, 1872 validation utterances of 6 s each, and 543 test utterances of 6 s each. The ratios of the training, validation, and test sets were 87%, 10%, and 3%, respectively. Signal-to-noise ratios (SNRs) ranged from −5 dB to 10 dB.

### 4.2. Model Configurations

All data were sampled at 16 kHz. To transform the data into the STFT domain, we used a Hanning window with a length of 512 and an overlap of 50%. We set the channel dimension length of the first Conv2D layer as D=48 and the number of feature integration blocks as N=3. The number of hidden units was set to $H_{1}=256$ for the first LSTM layer and $H_{2}=128$ for the second LSTM layer. The window size for the window partition operation was set to 8. The network was trained using the Adam optimizer [29] with a learning rate of 0.001 and a batch size of 2 input sequences. We trained our model using the same loss function as proposed in [30], and the epoch count was set to 150.

The performance of the model in this work was evaluated using the following three metrics: perceptual evaluation of speech quality (PESQ) [31], which evaluates objective speech quality; short-time objective intelligibility (STOI) [32], which evaluates objective speech intelligibility; and scale-invariant signal-to-distortion ratio (SI-SDR) [33]. The PESQ score ranges from −0.5 to 4.5, and the STOI score ranges from 0 to 1, with higher scores indicating better quality and intelligibility for both of these metrics.

## 5. Experimental Results and Analysis

### 5.1. Ablation Study

In this section, we performed an ablation study on the SPA-DNS dataset to analyze the individual contributions of each module mentioned above. We compared the results of using different fusion types, the presence of the GLAF module, and the number of feature integration blocks, as shown in Table 1. Table 1 details the number of parameters for each case, the number of floating point operations per second, the real-time factor (RTF) tested on an Intel(R) Xeon(R) Gold 5218R processor, sourced from Intel Corporation, Santa Clara, CA, USA, and the memory usage during the training step with a batch size of 1. The RTF is defined as the ratio of the time taken to process the audio to the duration of the audio itself. A lower RTF indicates better suitability for real-time applications, while a higher RTF suggests increased computational requirements, which may make real-time deployment more challenging. We utilized the Python toolbox profile to calculate the FLOPs. The Python version is 3.11.5, and the profile is imported from thop 0.1.1.

It can be observed that the FLOPs increased from 5.67 G/s in Case A to 17.09 G/s in Case E, while the memory usage increased from 8087 MB to 17,632 MB. Furthermore, there were consistent performance improvements from Case A to Case E, highlighting the effectiveness of the introduced module. This growth in model size and computation resulted in a longer training time. Additionally, the increased number of parameters imposes challenges for real-time deployment, particularly in resource-constrained environments. Therefore, practical deployment, especially on edge devices, requires careful consideration of the trade-offs between model complexity and performance gains.

The detailed analysis indicated the contribution of each module as follows:

- Full- and Sub-Band LSTM Module Contribution: Compared to the noisy input, Case A, which contained only two LSTM layers, showed significant improvements across all evaluation metrics. Specifically, the PESQ score improved from 1.56 to 2.77, the STOI score from 0.652 to 0.902, and the SI-SDR from −6.56 to 5.16, demonstrating the effectiveness in capturing spectral information. This module provided a foundational improvement by allowing the model to learn from both full- and sub-band spectral features, which are crucial for enhancing noisy speech.
- GLAF Module Contribution: Case A, which lacked the GLAF module, exhibited the poorest performance among all cases. The inclusion of the GLAF module in Case B resulted in gains of +0.009 in PESQ and +0.004 in STOI compared to Case A, highlighting the effectiveness of refining spectral information by incorporating global–local attention. This module plays an important role in enhancing specific details by applying both global and local attention, thereby effectively boosting the precision of the extracted features.
- Fusion Type Impact: A comparison between Case B and Case C showed that fusion using SA (spatial attention) outperformed the fusion by summation, resulting in improvements of +0.13 in PESQ, +0.013 in STOI, and +1.28 in SI-SDR. This improvement highlights that using spatial attention to selectively integrate feature information is more effective than a simple summation approach, leading to more significant enhancements in speech quality and intelligibility.
- Number of Feature Integration Blocks: Increasing the number of feature integration blocks from one (Case A) to three (Case E) consistently improved performance. Specifically, the PESQ score increased from 2.99 to 3.62, the STOI score from 0.919 to 0.965, and the SI-SDR from 6.20 to 12.00. These findings illustrate that having more feature integration blocks leads to better model performance, although at the cost of increased parameter size and computational complexity. The performance gains in terms of PESQ, STOI, and SI-SDR make the additional complexity worthwhile in scenarios where high-quality speech enhancement is crucial. However, in real-time or resource-constrained deployments, the increase in parameters and computational cost must be balanced against these improvements.

**Figure 4:**
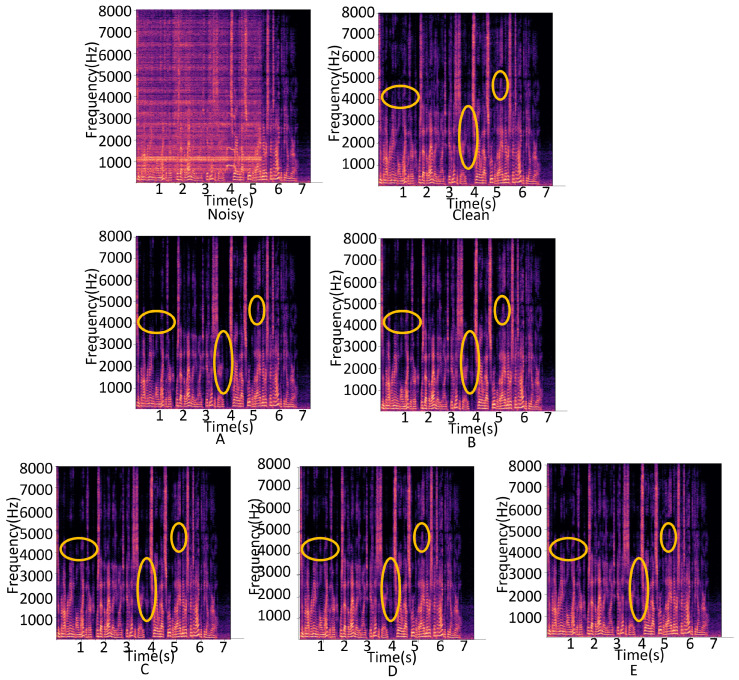
Spectrograms of the noisy, clean, and the five cases in Table 1 (**A**–**E**).

Figure 4 displays spectrograms of the noisy signal, the clean signal, and the cases presented in Table 1. The remaining spectrograms (from the top middle to the bottom row) illustrate the outputs from each model configuration, starting with Case A and progressing to Case E. As we move from Case A to Case E, the spectrograms reveal progressively more refined spectral details, with noise being more effectively suppressed in the later cases. Notably, the regions highlighted by yellow circles indicate areas where the model’s performance in recovering speech details improved. Case A contained relatively sparse spectral details due to the limited configuration of modules, resulting in less effective noise suppression. With each subsequent case, the integration of additional modules (such as the GLAF module and more feature integration blocks) led to noticeable improvements in the recovered spectral content. Case E, with the full model configuration, closely resembles the clean spectrogram, demonstrating the substantial enhancement achieved by our approach. The improvements shown in these spectrograms are consistent with the quantitative results in Table 1, indicating that each module contributed significantly to the enhancement in performance. As the value of *N* increased, the performance improved; however, the number of parameters and FLOPs also increased accordingly. A balance between model size and performance should be considered for practical deployment, especially on edge devices.

### 5.2. Comparison to the Baseline Models

In this section, we conducted a comparative analysis of our proposed multi-channel speech enhancement method against other state-of-the-art models using both the SPA-DNS and Libri-wham datasets. The SPA-DNS dataset was generated using a circular microphone array, whereas the Libri-wham dataset was generated using a linear microphone array. We compared our model with three non-causal advanced models: FasNet-TAC [34], an end-to-end speech enhancement neural network; EaBNet [10], a two-stage neural beamformer; and FT-JNF [7], a DNN-based joint non-linear filter. It is worth noting that while the datasets were simulated, the noise conditions and microphone setups were specifically chosen to replicate real-world scenarios, such as home and office environments, where speech enhancement systems are often deployed.

Table 2 presents the results of the models trained separately on the SPA-DNS and Libri-wham datasets. Our model was trained using the same configuration as for Case E in Table 1. It can be seen that our proposed model outperformed the three advanced models, whether using a circular microphone array or a linear microphone array. For the SPA-DNS dataset, which was generated using a circular microphone array, the PESQ score improved by 2.06, the STOI score by 0.313, and the SI-SDR score by 18.56. For the Libri-wham dataset, generated using a linear microphone array, the PESQ score improved by 1.99, the STOI score by 0.338, and the SI-SDR score by 20.816. These results demonstrate that our model effectively generalized across different microphone configurations, highlighting its robustness.

Compared to FasNet-TAC, which had the largest parameter size of 4.1 M, our model achieved better performance with fewer parameters (2.7 M for Case E). Despite having fewer parameters, our model achieved significant improvements across PESQ, STOI, and SI-SDR metrics, which demonstrated an efficient balance between parameter size and enhancement quality. This makes our model more suitable for deployment on devices with limited resources, where memory and computational power are critical constraints. Although FasNet-TAC had the most parameters, it performed worse on both the SPA-DNS dataset (+0.741 in PESQ, +0.172 in STOI, +10.881 in SI-SDR) and the Libri-wham dataset (+0.47 in PESQ, +0.098 in STOI, +7.276 in SI-SDR). Compared with EaBNet and FT-JNF, our proposed model stood out with its smaller parameter size and excellent performance.

The improvements observed in PESQ, STOI, and SI-SDR came with increased computational complexity, as evidenced by the growth in FLOPs and parameter count from Case A to Case E. This trade-off must be carefully considered in practical deployment scenarios, particularly those requiring real-time performance. In high-resource environments, such as powerful telecommunication servers, this increased complexity is acceptable, due to the significant gains in speech quality and intelligibility. However, in resource-constrained environments, such as battery-powered IoT devices, our model might require further optimization to maintain a balance between enhancement performance and computational feasibility. In conclusion, the results demonstrate that our proposed model consistently achieved the highest scores while maintaining the smallest parameter size, underscoring the effectiveness of our approach.

### 5.3. Robustness to Unseen Noise and Reverberation Time

To further analyze the generalization capability of our model in real-world noisy environments, we conducted additional experiments with both unseen noise and varying reverberation conditions. A test set was created consisting of unseen noise samples, which were real-life recordings of everyday noises obtained from Freesound. Table 3 presents the performance metrics of the various models trained on the Libri-wham dataset and evaluated on this unseen noise. Our proposed model (denoted as “Prop.”) achieved the highest performance across all evaluated metrics, with a PESQ of 3.54, a STOI of 0.951, and an SI-SDR of 10.08. Compared to the other models, these results demonstrate significant improvements in both speech quality and intelligibility, highlighting the effectiveness of our approach under unseen noise conditions. This superior performance indicates a strong generalization capability, making our model more robust for dealing with real-world noisy environments.

**Figure 5:**
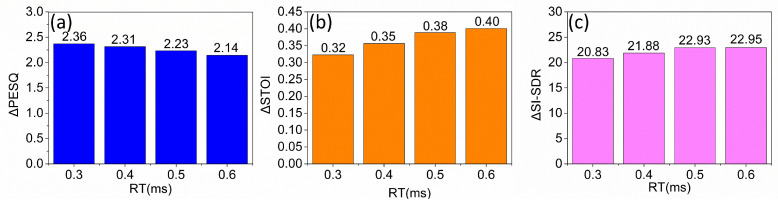
The influence of the reverberation time in terms of ΔPESQ, ΔSTOI, and ΔSI_SDR is shown in (**a**–**c**).

Furthermore, we evaluated our proposed model under several different reverberation conditions to assess its robustness further. Figure 5 illustrates the performance of our model in terms of improvements (Δ) in PESQ (Figure 5a), STOI (Figure 5b), and SI-SDR (Figure 5c) across various reverberation scenarios. The results demonstrate that our model consistently maintained a high performance, even with varying levels of reverberation, indicating resilience and adaptability.

Overall, the combined evaluation under unseen noise conditions and multiple reverberation levels provides strong evidence for the robustness of our proposed model. These findings suggest that our model can effectively generalize to challenging real-world conditions, which is crucial for practical applications in speech enhancement.

## 6. Conclusions

In this paper, we proposed a feature integration network for multi-channel speech enhancement, consisting of a Conv2D layer, several feature integration blocks, and a Deconv2D layer. Each feature integration block includes a full- and sub-band LSTM module, as well as a global–local attention fusion module. The experimental results demonstrated that each module significantly contributed to the overall enhancement performance. Additionally, the proposed model outperformed other state-of-the-art models in PESQ, STOI, and SI-SDR on both the SPA-DNS and Libri-wham datasets. In future work, we aim to enhance the model’s capability for practical applications, particularly in diverse and challenging real-world environments.

## Figures and Tables

**Table 1:** Experimental results of ablation study. The bold values show the best results.

Case	Para. (M)	FLOPs (G/s)	RTF	N	GLAF	Fusion Type	Memory Usage (MB)	PESQ	STOI	SI-SDR
Noisy	-	-	-	-	-	-	-	1.56	0.652	−6.56
A	0.85	5.67	0.12	1	×	-	8087	2.77	0.902	5.16
B	0.90	5.69	0.71	1	✓	sum	8091	2.86	0.906	4.92
C	0.91	5.70	0.72	1	✓	SA	8093	2.99	0.919	6.20
D	1.8	11.40	0.83	2	✓	SA	11,585	3.50	0.957	10.75
E	2.7	17.09	0.95	3	✓	SA	17,632	**3.62**	**0.965**	**12.00**

**Table 2:** Performance metrics of various models trained separately on the SPA-DNS dataset and Libri-wham dataset. The bold values show the best results.

Dataset	Model	Cau.	Para. (M)	PESQ	STOI	SI-SDR
SPA-DNS	Noisy	-	-	1.56	0.652	−6.56
	FasNet-TAC [34]	×	4.1	2.301	0.824	4.321
	EaBNet [10]	×	2.8	2.718	0.878	3.904
	FT-JNF [7]	×	3.3	2.886	0.885	5.269
	Prop.	×	2.7	**3.62**	**0.965**	**12.00**
Libri-wham	Noisy	-	-	1.44	0.604	−12.436
	FasNet-TAC [34]	×	4.1	1.91	0.702	−5.160
	EaBNet [10]	×	2.8	2.37	0.810	−2.292
	FT-JNF [7]	×	3.3	2.50	0.854	1.894
	Prop.	×	2.7	**3.43**	**0.942**	**8.38**

**Table 3:** Performance metrics of the various models trained on the Libri-wham dataset but tested on unseen noise. The bold values show the best results.

Model	PESQ	STOI	SI-SDR
Noisy	1.38	0.592	−12.11
FasNet-TAC [34]	1.72	0.677	−6.09
EaBNet [10]	2.23	0.784	−2.56
FT-JNF [7]	2.23	0.823	0.87
Prop.	**3.54**	**0.951**	**10.08**

## Data Availability

This work uses three open-source audio datasets (DNS Challenge 2020 corpus [26], Librispeech (train-clean-100) [27], and MUSAN dataset [28]. All open-source datasets are available online.
